# NF Erythroid 2-Related Factor 2 Peroxisome Proliferator-Activated Receptor-*γ* Crosstalk: Reprogramming Monocytic Myeloid-Derived Suppressor Cells in Kidney Disease

**DOI:** 10.34067/KID.0000001071

**Published:** 2025-12-02

**Authors:** John D. Imig

**Affiliations:** Department of Pharmaceutical Sciences, College of Pharmacy, University of Arkansas for Medical Sciences, Little Rock, Arkansas

**Keywords:** acute kidney failure, CKD, immunosuppression, ischemia-reperfusion, oxidative stress

The role of the immune system has been recognized as a major contributor to acute and CKD.^1^ Immune cells can respond can have both positive and negative contributions to kidney disease.^1^ The current report by Jia *et al*. explored the role of myeloid-derived suppressor cells (MDSCs), which are immature myeloid cells that limit T-cell responses.^2^ MDSCs exhibit two phenotypes, monocytic MDSCs (M-MDSCs) and granulocytic MDSCs (G-MDSCs), which have been implicated in cancer, organ transplantation, and chronic inflammatory diseases.^3^ In the experimental studies, Jia *et al*. determined the contribution of M-MDSCs and G-MDSCs to cancer, acute kidney disease, and CKD.^2^ Another aspect of the study explored NF erythroid 2-related factor 2 (Nrf2), which induces antioxidant gene expression in response to reactive oxygen species. The link between Nrf2 and MDSC-mediated immunosuppression was determined in mouse models of cancer and kidney diseases. Nrf2-deficient mice had enhanced immunosuppressive function of M-MDSCs which contributed to tumor progression. On the other hand, Nrf2 deficiency positively affected kidney diseases. A major finding was that Nrf2 deficiency enhances the immunosuppressive function of M-MDSCs and ameliorates acute kidney disease and CKD.

Although the last decade has seen a dramatic increase in approved drugs to treat kidney diseases, these treatments are not expected to curb the rise in kidney diseases and the need for organ transplantation.^4,5^ Major areas being explored for kidney disease treatments include immune cell regulation and the Nrf2 Kelch ECH associating protein 1 (Keap1) signaling pathway.^6^ Several immune cells including T cells have been extensively studied in kidney diseases and linked to Nrf2/Keap1 signaling.^6^ There is less known about MDSCs, their link to Nrf2/Keap1, and contribution to kidney diseases. This study evaluated the contribution of MDSCs in acute kidney ischemia reperfusion injury in mice. Mice had one kidney removed and exposed to 30 minutes ischemia followed injection of M-MDSCs or G-MDSCs from C57BL/6 or Nrf2^−/−^ mice. Interestingly, C57BL/6-MDSCs significantly attenuated the level of inflammatory cell infiltration into the kidneys and dilation of renal tubules, whereas Nrf2^−/−^ M-MDSCs had a more dramatic decrease in renal injury. Nrf2^−/−^ M-MDSCs had a large effect on CD4^+^ and CD8^+^ T-cell infiltration into the kidneys and lower proinflammatory cytokine TNF-*α* and IL-1*β* production. These studies demonstrate Nrf2-deficient M-MDSCs have a therapeutic action in acute kidney disease.

Next, studies were conducted in a mouse model of CKD to determine the contribution of Nrf2 and M-MDSCs. Mice were subjected to 5/6 nephrectomy and injected with M-MDSCs or G-MDSCs from C57BL/6 or Nrf2^−/−^ mice on days 0 and 7. Like AKI, C57BL/6 or Nrf2^−/−^ M-MDSCs decreased kidney damage at 1 month with Nrf2-deficient M-MDSCs demonstrating a greater decrease in kidney damage. T-cell infiltration and cytokine TNF-*α* and IL-1*β* production reduced which resulted in a dramatic decrease in renal fibrosis in the mice injected with the Nrf2-deficient M-MDSCs.

Cell signaling mechanisms were further explored in MDSCs. M-MDSCs from the bone marrow of Nrf2^−/−^ mice were induced to evaluate the cell signaling mechanisms by which Nrf2-deficient M-MDSCs exert kidney protective effects. A known mechanism for M-MDSCs is suppression of immune responses through inducible nitric oxide synthase and nitric oxide production.^7^ In the study by Jia *et al*.,^2^ induced M-MDSCs derived from Nrf2^−/−^ mice demonstrated increased levels of inducible nitric oxide synthase, whereas Arg1 levels were significantly downregulated. These data demonstrated that Nrf2^−/−^ M-MDSC-mediated immunosuppressive function was independent of Arg1 expression. Further RNA-seq data revealed strong correlation with peroxisome proliferator-activated receptor-*γ* (PPAR*γ*) for Nrf2-deficient M-MDSC. Similarly, PPAR*γ* blockade decreased the percentage of Nrf2-deficient M-MDSCs and blocked the suppression of T-cell proliferation. Finally, PPAR*γ* inhibition reversed the protective effect of Nrf2^−/−^ M-MDSCs in AKI and CKD. Taken together, these findings provide strong evidence that PPAR*γ* is central to the enhanced immunosuppressive function of Nrf2^−/−^ M-MDSCs, revealing a novel mechanism underlying MDSC differentiation.

The article concludes that the findings provide a solid foundation for the potential application of Nrf2-modified M-MDSCs to treat inflammatory acute kidney disease and CKD. Although the data are intriguing, several questions and gaps remain for a therapeutic approach to be realized. One major question for future studies is whether female mice would have decreased renal injury when injected with Nrf2^−/−^ M-MDSCs. There have been several studies that demonstrate sex differences in renal inflammation and degree of renal injury in mouse models of acute kidney disease and CKD.^8^ Another issue is the acute and CKD mouse models used in the studies. AKI involved the removal of one kidney rather than a bilateral warm ischemic AKI model. It is also important to determine if the renal protective findings with Nrf2^−/−^ M-MDSCs would extend to drug-induced models of AKI. The 5/6 nephrectomy mouse model used in this study likewise represents a severe model of CKD. CKD in humans occurs primarily due to hypertension and diabetes.^9^ Testing the anti-inflammatory and renal protective actions of Nrf2^−/−^ M-MDSCs in mouse models of hypertension and diabetes are warranted in the future. Another intriguing aspect of the study is that Nrf2 deficiency-mediated immunosuppressive mechanisms could be an underlying mechanism responsible for the transition of acute kidney disease to CKD. Previous studies have provided strong evidence for immune T-cell regulation in acute kidney disease progression to CKD.^1^ Additional studies will be needed to determine the role of Nrf2-deficient M-MDSCs in the transition from acute kidney disease to CKD. Overall, this study by Jia *et al*. is an exciting initial finding that Nrf2-deficient M-MDSCs has therapeutic implications in inflammatory acute kidney disease and CKD.^2^

Findings from this study by Jia *et al*. provide intriguing therapeutic potential for altering Nrf2 PPAR*γ* crosstalk in M-MDSCs in kidney diseases.^2^ The role of Nrf2^−/−^ G-MDSCs and Nrf2^−/−^ M-MDSCs on tumor size and tumor angiogenesis will complicate the potential therapeutic value of Nrf2-deficient M-MDSCs for kidney diseases. Interestingly, Nrf2 deficiency enhances the immunosuppressive function of M-MDSCs, which contributes to tumor progression. The central challenge moving forward is identifying strategies to target MDSCs while minimizing cancer risk. Novel therapeutic approaches that specifically target PPAR*γ* and Nrf2 on M-MDSCs are needed. Given the many advances in cell-specific drug delivery over the past decade,^10^ it is reasonable to envision the development of a therapeutic that targets Nrf2 PPAR*γ* crosstalk within M-MDSCs to treat both acute and CKD.
